# Inactivated SARS-CoV-2 induces acute respiratory distress syndrome in human *ACE2*-transgenic mice

**DOI:** 10.1038/s41392-021-00851-6

**Published:** 2021-12-24

**Authors:** Zhenfei Bi, Weiqi Hong, Haiying Que, Cai He, Wenyan Ren, Jingyun Yang, Tianqi Lu, Li Chen, Shuaiyao Lu, Xiaozhong Peng, Xiawei Wei

**Affiliations:** 1 Laboratory of Aging Research and Cancer Drug Target, State Key Laboratory of Biotherapy, National Clinical Research Center for Geriatrics, West China Hospital, Sichuan University, No. 17, Block 3, Southern Renmin Road, Chengdu, Sichuan 610041 PR China; 2 National Kunming High-level Biosafety Primate Research Center, Institute of Medical Biology, Chinese Academy of Medical Sciences and Peking Union Medical College, Kunming, Yunnan China

**Keywords:** Inflammation, Infection, Biological models, Infectious diseases

## Abstract

The development of animal models for COVID-19 is essential for basic research and drug/vaccine screening. Previously reported COVID-19 animal models need to be established under a high biosafety level condition for the utilization of live SARS-CoV-2, which greatly limits its application in routine research. Here, we generate a mouse model of COVID-19 under a general laboratory condition that captures multiple characteristics of SARS-CoV-2-induced acute respiratory distress syndrome (ARDS) observed in humans. Briefly, human *ACE2*-transgenic (*hACE2*) mice were intratracheally instilled with the formaldehyde-inactivated SARS-CoV-2, resulting in a rapid weight loss and detrimental changes in lung structure and function. The pulmonary pathologic changes were characterized by diffuse alveolar damage with pulmonary consolidation, hemorrhage, necrotic debris, and hyaline membrane formation. The production of fatal cytokines (IL-1β, TNF-α, and IL-6) and the infiltration of activated neutrophils, inflammatory monocyte-macrophages, and T cells in the lung were also determined, suggesting the activation of an adaptive immune response. Therapeutic strategies, such as dexamethasone or passive antibody therapy, could effectively ameliorate the disease progression in this model. Therefore, the established mouse model for SARS-CoV-2-induced ARDS in the current study may provide a robust tool for researchers in the standard open laboratory to investigate the pathological mechanisms or develop new therapeutic strategies for COVID-19 and ARDS.

## Introduction

The high mortality of SARS-CoV-2 infection is closely associated with the disease progression to a severe COVID-19 induced acute respiratory distress syndrome (ARDS).^1^ The mortality rate in COVID-19 ARDS patients is up to 50% and reaches 94% in those who received mechanical ventilation.^2^ The lethal features include increased leukocyte infiltration in the lungs, hyperinflammatory responses, thick mucus secretion in the airways, and microthrombosis, leading to the diffuse alveolar damage and extensive pulmonary injury.^3,4^ However, the mechanisms underlying how the host response towards SARS-CoV-2 results in ARDS and severe COVID-19 illness remains unclear. The high infectious rate of SARS-CoV-2 and increased mortality caused by ARDS have put an enormous strain on the health care system all over the world. Despite the preventative vaccines, more therapeutic strategies are urgently needed.

Animal models are essential for understanding viral pathogenesis and finding new therapeutic targets. Importantly, proper models are vital for accelerating the development of vaccines and antiviral drugs. To date, several animal species, including non-human primates, hamsters, mice, ferrets, civet cats, and rabbits, have been well utilized in the studies for SARS-CoV-2;^5^ however, these models could not fully recapitulate the characteristics of severe lung disease observed in humans.^5^ SARS-CoV-2 utilizes the cellular receptor human angiotensin-converting enzyme 2 (*hACE2*) to enter the host cells, but not the mouse *ACE2* (*mACE2*).^6^ Thus, infection of laboratory strains of mice requires adapted viruses by serial passage,^7,8^ or introducing *hACE2* into mice via adenoviral/adenoviral-associated vectors,^9^ mouse *ACE2* promoter,^10^ or heterologous gene promoters.^11^ At present, various categories of vaccines, such as mRNA vaccines or recombinant protein vaccines,^7,12–16^ and small molecular drugs for COVID-19 are still under evaluation in mouse models,^17^ however, most of the animal models need to be performed under high biosafety level conditions. Here, we propose that if the mouse COVID-19 model could be established under a normal laboratory condition and fully recapitulate the severe pathological phenotypes would be helpful for the preclinical evaluation of vaccines and drugs.

In the present study, we developed a mouse model for severe COVID-19 by intratracheal instillation of formaldehyde-inactivated SARS-CoV-2 virus (FA-S) in *hACE2* transgenic mice under a general laboratory condition. The pulmonary diseases induced by FA-S in *hACE2* mice were highly like the clinical symptoms and pathological changes of COVID-19 patients with ARDS. Moreover, the FA-S virus could active the adaptive immune response which occurs in late SARS-CoV-2 infection. We selected dexamethasone and passive antibody therapy as strategies for in vivo evaluation to demonstrate that the FA-S-induced model could be utilized in preclinical evaluation of drug candidates. This study may provide a practical mouse model that recapitulates the pathological features of SARS-CoV-2-induced ARDS in humans, which is easy to be established under standard laboratory conditions. It might also be helpful to develop a novel platform in vaccine and drug screening for SARS-CoV-2.

## Results

### *hACE2* mice are highly susceptible to intratracheal instillation of inactivated SARS-CoV-2

**Fig. 1:**
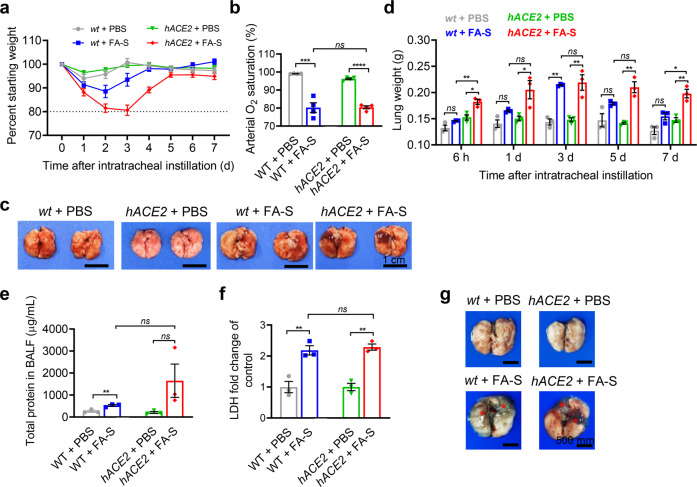
Intratracheal instillation of inactivated SARS-CoV-2 causes pulmonary injury in *hACE2* mice **a** Weight changes of *hACE2* and *wt* mice after instillation of PBS or inactivated SARS-CoV-2. *hACE2* mice treated with inactivated SARS-CoV-2 exhibited an approximately 20% loss of body weight on 3 dpi. **b** Detection of arterial O_2_ saturation on 3 dpi. **c**, **d** Images of lung tissues (**c**) and measurements of lung weight (**d**). **e**, **f** Total protein (**e**) and fold change of LDH release (**f**) in BALF. *wt* and *hACE2* mice treated with PBS were used as control. **g** Images of lung tissues with Evans blue dye retention on 3 dpi. Red asterisks indicated increased permeability appeared after instillation with inactivated SARS-CoV-2. *hACE2* mice developed pulmonary edema and severe lung injury after intratracheal instillation of inactivated SARS-CoV-2. Data represent the mean ± SEM. Significance is indicated by: *ns*, no significance; *P ≤ 0.05, **P ≤ 0.01, ***P ≤ 0.001, ****P ≤ 0.0001

We inoculated 10-week-old *hACE2* mice or wild type (*wt*) mice via intratracheal instillation with 2 × 10^^6^ plaque-forming units (PFU) of inactivated SARS-CoV-2. The control mice received the equal volume vehicle of PBS. Clinical symptoms and body-weight changes were monitored daily. Beginning at 1-day post instillation (dpi), mice demonstrated rapid weight loss, and reached the maximum weight loss on 2 dpi (*wt* mice) or on 3 dpi (*hACE2* mice) (Fig. 1a). The *hACE2* mice have more susceptibilities with an approximately 20% loss of body weight on 3 dpi (Fig. 1a). Moreover, significantly low levels of activity, mentality, and response to the stimulation were found in *hACE2* mice after instillation of inactivated SARS-CoV-2. All treated animals recovered on 7 dpi (Fig. 1a). Interestingly, in a *hACE2* mouse model, we intratracheally instilled *hACE2* mice with live SARS-CoV-2 and found the same trend in body-weight changes compared to that of *hACE2* mice instilled with inactivated SARS-CoV-2 in this study (Supplementary Fig. S1a).

Further, we also evaluated the details of oxyhemoglobin saturation in mice. Instillation of inactivated SARS-CoV-2 caused a marked deterioration in parameter of arterial O_2_ saturation on 3 dpi, with a decrease of almost 20% in *wt* and *hACE2* mice (Fig. 1b). The result was almost consistent with the clinical course of ARDS patients^18,19^ and other severe models of *hACE2* mice infected by SARS-CoV-2 or mice infected by mouse adapted SARS-CoV-2.^8,20,21^

### Inactivated SARS-CoV-2 causes acute respiratory distress syndrome (ARDS) in *hACE2* mice

To investigate whether the mouse model in this study recapitulates the pathologies observed in COVID-19 ARDS patients, we examined the lung tissues at 6 h, and on 1, 3, 5, and 7 days after instillation of inactivated SARS-CoV-2. We found the induction of the lungs of the bigger size, with the patches of dark-colored hemorrhage, and bilateral congestion and edema in *hACE2* mice treated with inactivated SARS-CoV-2 (Fig. 1c). We noted that the lung weight reached to the peak on 3 dpi both in treated *hACE2* mice and *wt* mice, and recovered to the control level on 7 dpi only in treated *wt* mice (Fig. 1d). Associated with these findings, elevated protein and LDH release in bronchoalveolar lavage fluid (BALF), and Evans blue dye retention in lung tissues of treated *hACE2* mice were observed on 3 dpi (Fig. 1e–g), indicating the increased vascular permeability and pulmonary injury caused by inactivated SARS-CoV-2.

**Fig. 2:**
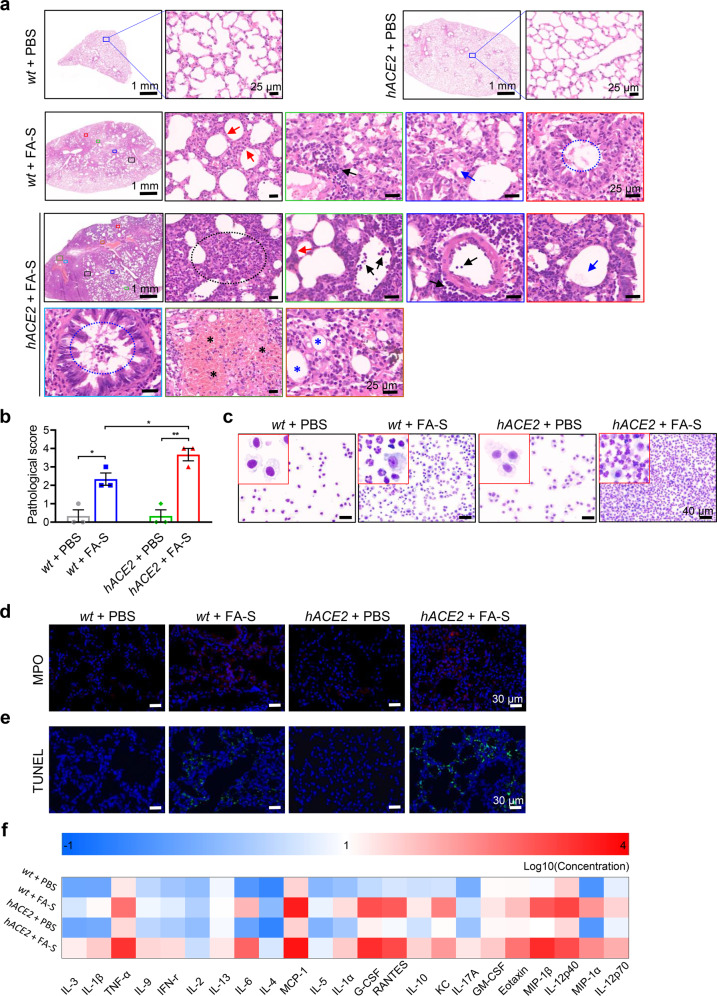
Inactivated SARS-CoV-2 induces ARDS in *hACE2* mice. **a** Images of H&E staining on 3 dpi. Immune cell infiltrations were found throughout the lung tissues in *hACE2* mice after instillation of inactivated SARS-CoV-2, including neutrophils, inflammatory monocyte-macrophages and T cells (black arrows and black dotted circle), with alveolar septal thickening (red arrow), proteinaceous (blue arrow), inflammatory cells and proteinaceous debris in bronchioles (blue dotted circle), hemorrhage with the red blood cells filling the alveolar spaces (black asterisks), hyaline membranes formation (blue asterisks), and interstitial edema and consolidation (black dotted circle). The magnified images of groups of *wt* + FA-S and *hACE2* + FA-S were indicated which area was extracted from the image with low magnification by the individual color border, respectively. **b** Pulmonary pathological scores for mice on 3 dpi according to the 5-grade scoring system. **c** Images of Diff-quik staining of cells in BALF on 3 dpi. **d**, **e** Images of immunofluorescence analysis for MPO (**d**) and TUNEL (**e**) in lung tissues on 3 dpi. **f** Screening and analysis of cytokines or chemokines in BALF by a customized Luminex Mouse Cytokine 23-plex. *hACE2* mice showed higher levels of cytokines production. Results were presented as log10 of the concentration. Data represent the mean ± SEM. Significance is indicated by *P ≤ 0.05, **P ≤ 0.01

Analysis of hematoxylin and eosin-stained (H&E) lung sections showed a progressive inflammatory process. At 6 h post instillation of inactivated SARS-CoV-2, we observed obvious accumulation of immune cells confining predominantly to perivascular sites, especially in *hACE2* mice (Supplementary Fig. S2b). By 1 dpi, the immune cells infiltrated into parts of lung tissues with alveolar septal thickening (Supplementary Fig. S2b). By 3 dpi, both treated *hACE2* and *wt* mice developed interstitial pneumonia characterized by inflammatory cell infiltration, interstitial edema, and consolidation (Fig. 2a). Pulmonary inflammation is a prominent manifestation of ARDS, and neutrophils in BALF correlate with the disease severity.^22,23^ We found a markedly elevated number of neutrophils around airway and perivascular sites, infiltrating to the pulmonary parenchyma and to the alveolar with increased MPO activities on 3 dpi, especially in treated *hACE2* mice (Fig. 2a, c, d). Neutrophils remained predominant cell infiltration throughout the lung tissues of both treated *hACE2* and *wt* mice (Fig. 2a). Obvious disruption of lung tissues and the disappearance of recognizable architecture could be easily observed in mice treated with inactivated SARS-CoV-2, and large number of dead cells were found in the background of inflammatory infiltrates (Fig. 2a). Of note, treated *hACE2* mice showed an earlier response and more severity pathological index with lung injury than those of *wt* mice (Supplementary Fig. S2a–c). In addition, hyaline membranes formation, necrotic debris, hemorrhage, proteinaceous debris, and apoptotic cells were also observed in treated *hACE2* mice on 3 dpi (Fig. 2a–e), consistent with diffuse alveolar damage. These results shared many features with those of a *hACE2* mouse model intratracheally instilled with live SARS-CoV-2 (Supplementary Fig. S1b). To better evaluate the pulmonary pathologies, we used a 5-grade scoring system as described previously.^20^ The *wt* mice treated with inactivated SARS-CoV-2 reached an average score of 2–3 on 3 dpi, and treated *hACE2* mice reached score of 3–4 (Fig. 2b). By 7 dpi, all treated animals showed alleviative pulmonary pathologies (Supplementary Fig. S2b). Together, these findings in treated *hACE2* mice well recapitulate the pathologies observed in COVID-19 ARDS patients^24,25^ and other animal ARDS models, coincident with enhanced lung weight, increased permeability, hemorrhage, edema, and severe pathological changes.^8,20,21^ Therefore, our mouse model may reflect the pathogenesis of SARS-CoV-2-induced ARDS in humans.

### The inflammatory injury induced by inactivated SARS-CoV-2 instillation

The host immune responses, including severe pro-inflammation with elevated cytokines in sera and BALF, particularly IL-6, IL-1β, and TNF-α, and impaired interferon responses, are markers of severe disease, manifesting as ARDS in some patients with COVID-19.^2,26–28^ Here, we investigated how the cytokine levels changed in mice followed by the inactivated SARS-CoV-2 instillation. Proinflammatory cytokines, such as IL-6, IL-1β, TNF-α, IL-2, IL-10, and G-CSF, showed robust increases both in sera and BALF on 3 dpi in treated mice (Fig. 2f and Supplementary Fig. S3). Moreover, chemokines including MCP-1, RANTES, Eotaxin, KC, and MIP-1α/β that recruit neutrophils, monocytes, macrophages or lymphocytes, were all elevated (Fig. 2f and Supplementary Fig. S3). In line with our pulmonary pathological analysis, much higher levels of cytokines in *hACE2* mice were observed than those in *wt* mice, suggesting the vital role of gene *hACE2* in the immune response in SARS-CoV-2 infection. These data are coincided with the cytokine profiling in sera or BALF from the COVID-19 patients.^27,28^

**Fig. 3:**
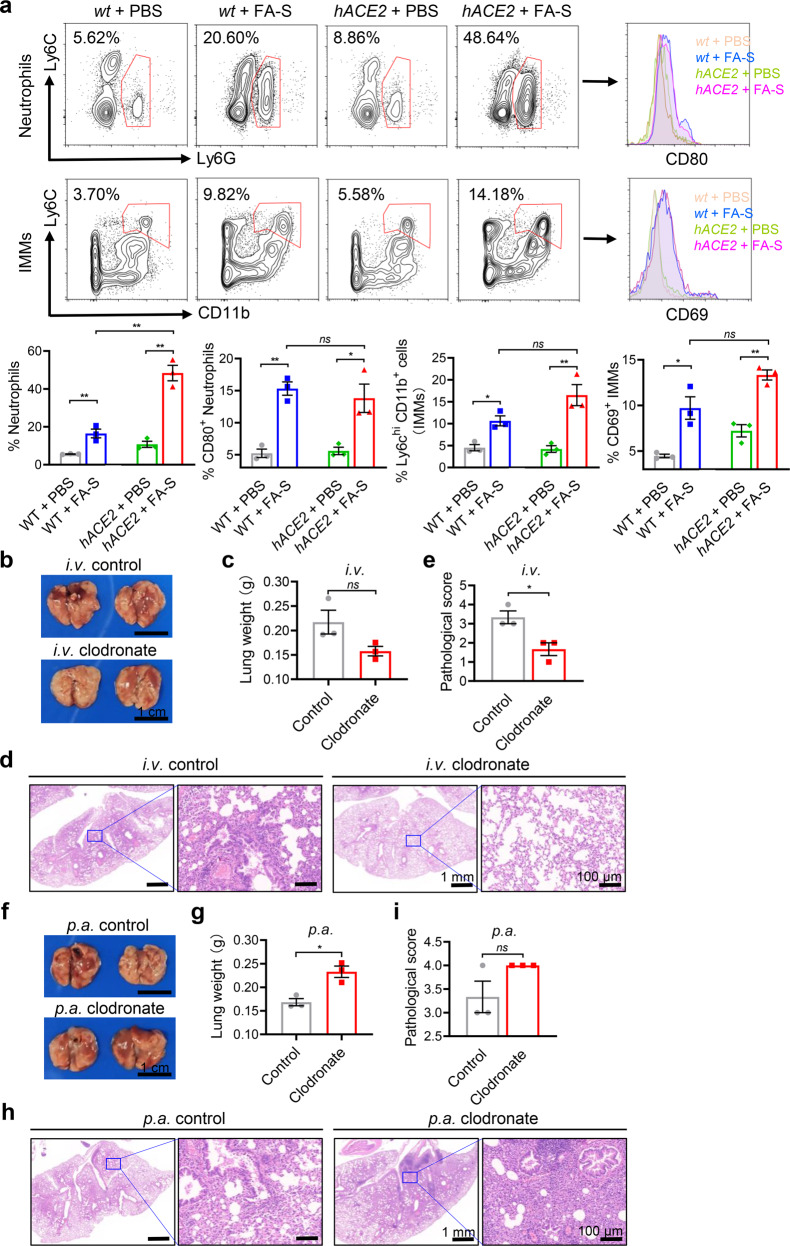
IMMs contribute to the progress of ARDS induced by inactivated SARS-CoV-2. **a** Flow cytometric analysis of neutrophils (labeled as Ly6G^+^), inflammatory monocyte-macrophages (IMMs, labeled as CD11b^+^ Ly6C^hi^) accumulations in lung tissues and correlated levels of activation markers (CD69 and CD80) on 3 dpi. The treated *hACE2* mice showed high levels of pulmonary infiltration of activated neutrophils and IMMs. **b**–**e** Elimination of IMMs by *i.v*. injection of clophosome-A showed decreased lung weight and ameliorative pulmonary pathology, including lower interstitial edema, inflammatory cell infiltration, and pulmonary injury, in treated *hACE2* mice. Images of lung tissues (**b**), measurement of lung weight (**c**), images of H&E staining (**d**) and pulmonary pathological scores for mice according to the scoring system (**e**) on 3 dpi. **f**–**i** Elimination of pulmonary resident macrophages by *p.a*. administration of clophosome-A showed an exacerbated pulmonary pathology in treated *hACE2* mice. Images of lung tissues (**f**), lung weight (**g**), images of H&E staining (**h**) and pulmonary pathological scores for mice according to the scoring system (**i**) on 3 dpi. *i.v*., intravenous; *p.a*., pharyngeal aspiration. Data represent the mean ± SEM. Significance is indicated by: *ns*, no significance; *P ≤ 0.05, **P ≤ 0.01

Next, we performed flow cytometric analysis on lung homogenates and collected hemocytes on 3 dpi. Both treated *hACE2* mice and *wt* mice showed significantly increased recruitments of Ly6G^+^ neutrophils and CD11b^+^ Ly6C^hi^ inflammatory monocyte-macrophages (IMMs) to lung tissues (Fig. 3a); however, only treated *hACE2* mice showed elevated neutrophils in peripheral blood, indicating persistent chemotaxis (Supplementary Fig. S4). In addition, we also observed an increase in activation markers of CD69 in IMMs and CD80 in neutrophils^29^ (Fig. 3a). The IMMs accumulation is highly correlated with the severe pulmonary injury reported in MERS-CoV, SARS-CoV, and SARS-CoV-2 infections.^29–31^ Elimination of IMMs by intravenous (*i.v*.) injection of clophosome-A showed decreased lung weight and ameliorative pulmonary pathology, including lower interstitial edema, inflammatory cell infiltration, and pulmonary injury, with an average score of 1–2 in *hACE2* mice by inactivated SARS-Cov-2 instillation (Fig. 3b–e); however, elimination of pulmonary resident macrophages by pharyngeal aspiration (*p.a*.) administration of clophosome-A showed an exacerbated pulmonary pathology and an average score of 4 (Fig. 3f–i), suggesting the vital role of resident macrophages in the virus defense.

**Fig. 4:**
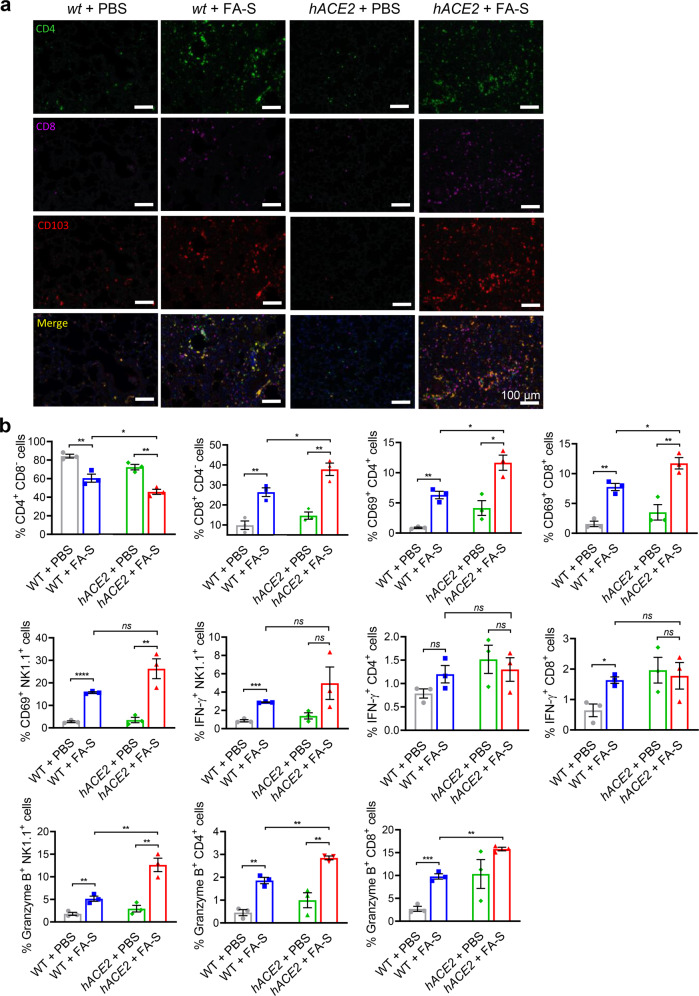
Lymphopenia is caused by inactivated SARS-CoV-2 in the lungs. **a** Immunofluorescence analysis of T cell accumulation in lung tissues on 3 dpi. Inactivated SARS-CoV-2 induced marked infiltration of T cells labeled as CD4- or CD8-positive with elevated CD103 expression. The treated *hACE2* mice showed more CD8-positive T cell infiltration. Green, CD4; violet, CD8; red, CD103; blue, DAPI. **b** Flow cytometric analysis of lymphocytes in lung tissues on 3 dpi. The percentage of CD8-positive T cells were markedly increased in CD3-positive T cells. Inactivated SARS-CoV-2 induced activation of CD4-positive T cells, CD8-posituve T cells and NK cells (labeled as CD69^+^) with elevated expressions of IFN-γ and Granzyme B. Data represent the mean ± SEM. Significance is indicated by: *ns*, no significance; *P ≤ 0.05, **P ≤ 0.01, ***P ≤ 0.001, ****P ≤ 0.0001

Further, we enumerated NK and T cells in peripheral blood and found a significant decrease in treated *hACE2* mice (Supplementary Fig. S4). Lymphopenia has been associated with severe COVID-19 in patients^32^. Of note, T cells showed more infiltrations to lung tissues with elevated CD103 expression and the percentage of CD8^+^ in CD3^+^ T cells was markedly elevated in treated *hACE2* mice (Fig. 4a, b). The activation marker of CD69 was increased in NK, CD4^+^ T and CD8^+^ T cells (Fig. 4b). The effectors of Granzyme B and IFN-γ were enhanced in NK and CD4^+^ cells; however, the CD8^+^ T cells in treated *hACE2* mice did not show increased secretion of Granzyme B or IFN-r (Fig. 4b). Overall, these results demonstrate the accumulation of activated neutrophils and IMMs, and lymphocytes in lung tissues after inactivated SARS-CoV-2 instillation, suggesting an injurious innate inflammatory response, especially in *hACE2* mice.

### Inactivated SARS-CoV-2 induces an adaptive immune response in *hACE2* mice

**Fig. 5:**
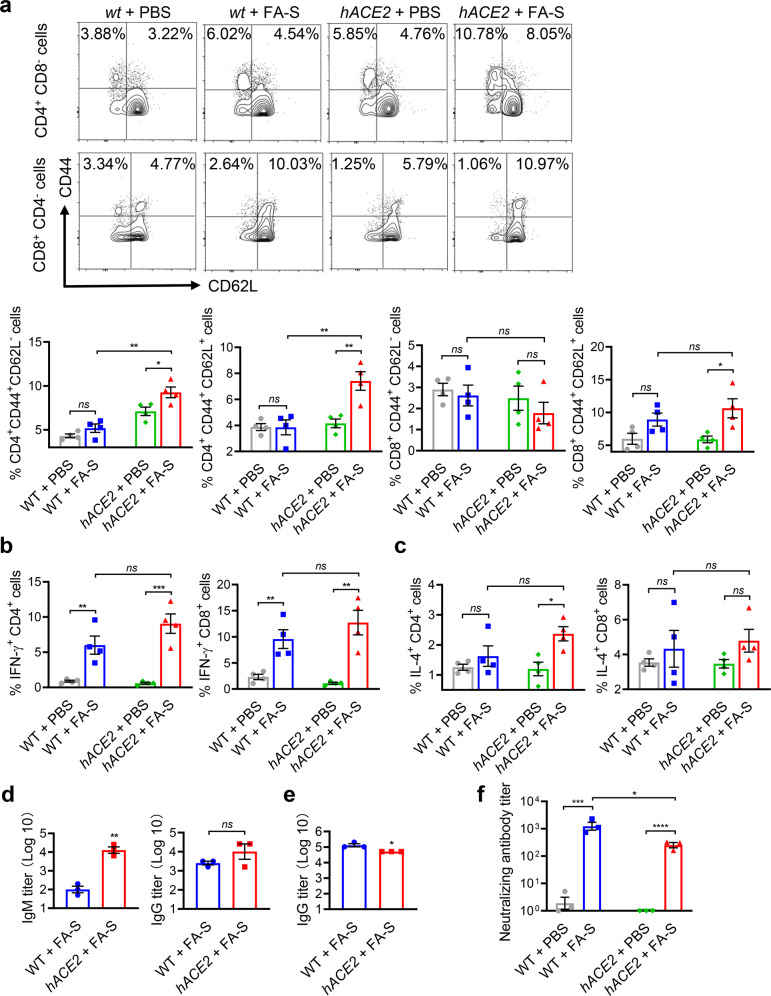
Inactivated SARS-CoV-2 induces an adaptive immune response in *hACE2* mice. **a** Flow cytometric analysis of subsets of effector (labeled as CD44^+^ and CD62L^-^) and central (labeled as CD44^+^ and CD62L^+^) memory T cells from inguinal lymph nodes in mice on 14 dpi. **b**, **c** Flow cytometric analysis on the secretion of IFN-γ (**b**) and IL-4 (**c**) in T cells from spleens on 14 dpi when stimulated with recombinant S1 protein. **d**, **e** Detection of IgG and IgM antibodies titers in sera against the S1 protein of SARS-CoV-2 on 7 dpi (**d**) and 14 dpi (**e**). **f** Detection of pseudovirus neutralization activity of the sera on 14 dpi. Data represent the mean ± SEM. Significance is indicated by: *ns*, no significance; *P ≤ 0.05, **P ≤ 0.01, ***P ≤ 0.001, ****P ≤ 0.0001

To investigate the adaptive immune response involved in the clearance of the inactivated SARS-CoV-2, we isolated T cells from spleens and inguinal lymph nodes in mice on 14 dpi. Subsets of effector (CD44^+^ CD62L^-^) and central (CD44^+^ CD62L^+^) memory CD4^+^ T cells were significantly increased; however, only subset of central memory CD8^+^ T cells increased in inguinal lymph nodes of treated *hACE2* mice, manifesting a robust adaptive immune response in *hACE2* mice post instillation of inactivated SARS-CoV-2 (Fig. 5a and Supplementary Fig. S5a). Further, we cultured T cells from spleens in mice on 14 dpi and performed flow cytometric analysis on the secretion of IFN-γ and IL-4. Elevated levels of IFN-γ and IL-4 in T cells when stimulated with recombinant RBD, S1 or S protein in treated *hACE2* mice were observed; however, treated *wt* mice only showed an elevated level of IFN-γ in T cells when stimulated with S1 or S protein, and no significant change or a decrease of IL-4 level when stimulated with either RBD, S1 or S protein (Fig. 5b, c and Supplementary Fig. S5b, c).

Convalescent patients of COVID-19 have high antibody titers in their serum, which could neutralize the activity of the SARS-CoV-2 virus.^33^ Thus, we collected sera from mice treated with PBS or PF-S to ensure whether humoral immunity could be induced by inactivated SARS-CoV-2. Sera obtained from treated *hACE2* mice showed elevated IgM and IgG responses to the S1 protein of SARS-CoV-2 on 7 dpi (Fig. 5d), and even higher level of IgG to S1 protein on 14 dpi (Fig. 5e). By contrast, the sera from control mice treated with PBS showed only background-level antibody responses. In addition, we also assessed the viral neutralization activity of the sera on 14 dpi challenged in vitro with a pseudovirus, and high levels of activity were observed both in *hACE2* mice and *wt* mice (Fig. 5f). These findings suggested that instillation of inactivated SARS-CoV-2 could induce a strong adaptive immune response, including cellular and humoral immunity in *hACE2* mice.

### Inactivated SARS-CoV-2 allows rapid evaluation of drugs and vaccines

**Fig. 6:**
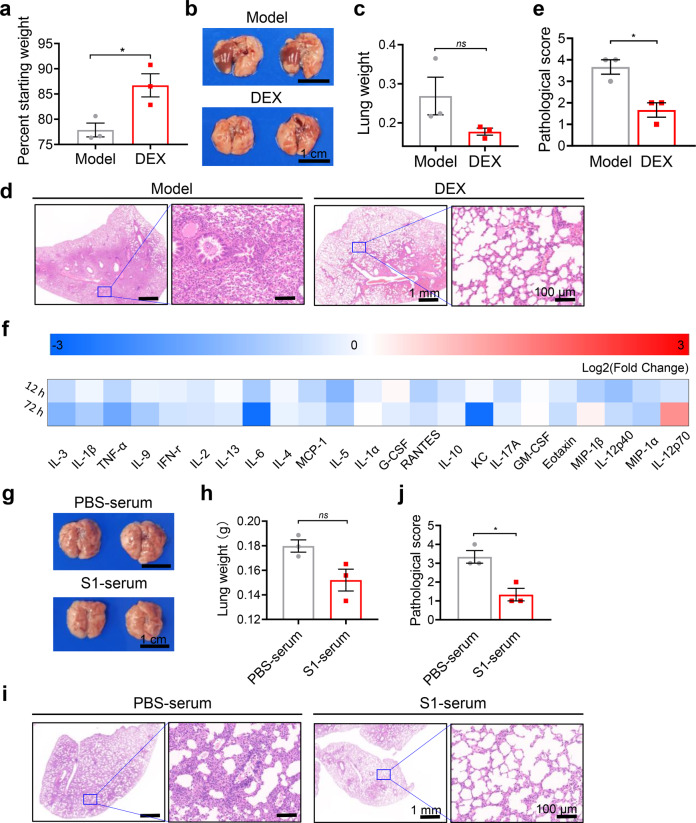
Dexamethasone and passive antibody therapy alleviated the progress of ARDS induced by inactivated SARS-CoV-2 in *hACE2* mice. **a**–**f** Reduced body-weight loss (**a**), images of lung tissues (**b**), measurement of lung weight (**c**), images of H&E staining (**d**), pulmonary pathological scores for mice according to the scoring system (**e**) and analysis of cytokines or chemokines in sera by a customized Luminex Mouse Cytokine 23-plex (**f**) on 3 dpi in *hACE2* mice when treated with dexamethasone. **g**–**j** Images of lung tissues (**g**), measurement of lung weight (**h**), images of H&E staining (**i**) and pulmonary pathological scores for mice according to the scoring system (**j**) on 3 dpi in *hACE2* mice when passively immunized with sera from S1 protein immunized mice. Data represent the mean ± SEM. Significance is indicated by: *ns*, no significance; *P ≤ 0.05

To explore the potential in the evaluation of drugs for COVID-19 ARDS, we administrated the mouse model with dexamethasone, a recognized effective drug for COVID-19 ARDS.^34^ We noted that treatment with dexamethasone significantly reduced the body-weight loss (Fig. 6a) and the lung weight (Fig. 6c) in *hACE2* mice on 3 dpi. Analysis of H&E lung sections showed alleviated pathological changes, including decreased inflammatory cell infiltration, lower interstitial edema, and necrotic debris with an average score of 1–2 compared to the ARDS mouse model (Fig. 6b–d, e). Moreover, we detected cytokine levels after dexamethasone treatment. Proinflammatory cytokines and chemokines, such as IL-6, IL-1β, TNF-α, IL-2, IL-10, G-CSF MCP-1, RANTES, Eotaxin, KC, and MIP-1α/β, were markedly decreased compared to those in the ARDS mouse model on 3 dpi (Fig. 6f).

In addition, we also evaluated the vaccine efficacy of this mouse model. We passively immunized *hACE2* mice with sera from S1 protein immunized mice and showed effective protection against the inactivated SARS-CoV-2. Decreased lung weight and alleviated pathological changes were founded in *hACE2* mice treated with sera from S1 immunized mice and coincided with an average score of 1–2 compared to the ARDS mouse model (Fig. 6g–j). Therefore, we suggest that this inactivated SARS-CoV-2-induced mouse ARDS model is sensitive to drug treatment and may be applicable to drug screening and vaccine evaluation.

## Discussion

Animal models that can reproduce the features exhibited in COVID-19 ARDS patients are urgently needed. In this study, we reported a mouse ARDS model established by intratracheal instillation of the formaldehyde-inactivated SARS-CoV-2 in *hACE2* mice. We found that instillation of inactivated SARS-CoV-2 caused rapid weight loss in *hACE2* mice and induced severe pulmonary disease on 3 dpi. The inactivated SARS-CoV-2 has no infected ability but can induce intense inflammatory responses after immediate instillation. The adaptive immune response in mice was characterized by rapidly high levels of proinflammatory cytokines/chemokines and the infiltration of activated neutrophils and IMMs in lung tissues, and T cell activation and effector and central T cells production resulting in pulmonary interstitial edema and consolidation. This caused detrimental changes in lung structure and function, including decreased oxyhemoglobin saturation and stiffening of the lung parenchyma, and progressed to ARDS.

Understanding the immune signature in human SARS-CoV-2 infection is crucial in the development of new therapeutic targets to reduce the morbidity of SARS-CoV-2-induced ARDS. In the current study, we observed robust increases of proinflammatory cytokines/chemokines in sera and BALF, such as TNF-α, IL-1β, IL-6, KC, MCP-1, RANTES, and MIP-1α/β^27,28^ which correlate with those observed in severe COVID-19 and ARDS patients. The improper activation of neutrophils contributes to the pathogenesis of COVID-19 and ARDS in severe cases because of the high levels of ROS, NET production, diffuse microvascular thrombosis, and cytokine storm.^23,35,36^ In addition, the accumulation of IMMs, the pulmonary injury, and the impaired immune response were highly coincided with those in infections caused by influenza virus,^37^ MERS-CoV,^29^ SARS-CoV,^30^ and SARS-CoV-2.^31^ Notably, the ARDS induced by SARS-CoV-2 infection shares multiple similar immune signatures with that induced by influenza virus.^1^ Here, we saw substantial myeloid cell infiltration throughout the lung tissues in treated *hACE2* mice, including neutrophils, IMMs as well as activated T cells, with elevated MPO levels. The excess influx of neutrophils to alveolus pulmonis was also verified, which is highly related to ARDS development;^22,36^ however, this mouse model developed no obvious microvascular thrombosis. Pulmonary resident macrophages, i.e., alveolar macrophages, have an important role in maintaining pulmonary immune homeostasis and defending pathogen infections.^38^ Elimination of resident macrophages induced more severe pulmonary pathology,^39^ which was also confirmed in the current model. Of note, we observed a significant decrease of peripheric lymphocytes following instillation of inactivated SARS-CoV-2, consistent with lymphopenia observed in COVID-19 patients with ARDS.^32^ The immunological process caused severe pulmonary pathology with diffuse alveolar damage in this mouse model, exhibiting hyaline membrane formation, the collapse of the alveolar space, hemorrhage, and interstitial edema, *etc*. This course well coincides with human pulmonary pathological changes observed in SARS-CoV-2-induced ARDS.^24,25^ Moreover, we also demonstrated a robust production of antibodies and the strong pseudovirus-neutralizing activity associated with a humoral response. The antibodies in *hACE2* mice show great affinities to S1 protein of SARS-CoV-2, consistent with the observations in convalescent patients.^33^ The current mouse model is also well suitable to evaluate the medical countermeasures, such as dexamethasone and passive antibody therapy in this study.

Of note, SARS-CoV-2-induced endocytic loss of ACE2 at the cell membrane causes detrimental effects, followed by the overactivation of RAS and p38 MAPK signaling and inflammation.^40^ The combined responses further promote the infection of SARS-CoV-2 in humans with COVID-19.^40,41^ In addition, proteins from SARS-CoV-2 may directly upregulate p38 MAPK signaling and the following inflammation.^41^ Previous studies also demonstrated that intratracheal instillation of SARS-CoV-2 spike protein or spike protein subunit S1 could induce the pulmonary inflammatory response and COVID-19-like acute lung injury in *hACE2* mice.^42,43^ In our mouse model, *hACE2* mice exhibited more severity in pulmonary pathology and immune response than those of *wt* mice. We speculate that inactivated SARS-CoV-2 and components per se, especially spike glycoprotein, may be easier to combine with targeted cells via interacting with *hACE2*, which may help inactivated virus stay longer and trigger more intense inflammation. This may contribute the majority to the progress of the severe disease in *hACE2* mice instilled with inactivated SARS-CoV-2. Further studies on the inactivated SARS-CoV-2-induced pulmonary pathogenesis in *hACE2* mice are under investigation.

In summary, we found that *hACE2* mice are susceptible to intratracheal instillation of inactivated SARS-CoV-2 and exhibit severe immune cell infiltration, cytokine storm, and pulmonary disease. The pulmonary pathological changes observed in *hACE2* mice well resembled those observed in humans with COVID-19 ARDS. Therefore, the COVID-19 mouse model in the current study might be a useful animal model for studying the pathogenesis of severe ARDS, and evaluating vaccines and antiviral agents to combat SARS-CoV-2.

## Materials and methods

### Ethics and biosafety statement

All animal studies carried out were approved by the Animal Care and Use Committee of Sichuan University (Chengdu, Sichuan, China). Transgenic *hACE2* mice were approved by the Animal Care and Use Committee, the Institute of Laboratory Animal Science, Peking Union Medical College, China. All procedures involved in the live SARS-CoV-2 were reviewed and approved by the Institutional Animal Care and Use Committee of Institute of Medical Biology, Chinese Academy of Medical Science, and performed in the ABSL-4 facility of Kunming National High-level Biosafety Primate Research Center, Yunnan, China.

### Preparation of formaldehyde-inactivated SARS-CoV-2 virus (FA-S)

The SARS-CoV-2 isolate GD108# strain used in this study was initially from Guangdong Provincial Center for Disease Control and Prevention. Stocks were amplified in Vero E6 cell line monolayers maintained in DMEM medium (Gibco, USA). Infectious units were quantified by plaque assay. Formaldehyde-fixed SARS-CoV-2 (FA-S) was performed at 4 °C in the ABSL-4 facility. Formaldehyde in FA-S was removed by ultrafiltration using a regenerated cellulose membrane with 3 kDa NMWCO (Millipore, USA), then FA-S was resuspended in PBS and kept at 4 °C for further use.

### Mice and experiment protocol

A total of 10-week-old male C57BL/6 mice were purchased from Beijing Vital River Laboratory Animal Technology Company, and 10-week-old male *hACE2* C57BL/6 mice were obtained from the National Institutes for Food and Drug Control.^11^ The mice were housed in a specific pathogen-free environment. After acclimation, mice were anesthetized with 5% isoflurane and 2 × 10^^6^ PFU of PA-S (equal quantity to SARS-CoV-2) in 50 µl of PBS per mouse was intratracheally instilled with a 29-gauge insulin syringe. The vehicle control received an equal volume of PBS. Weight was monitored daily, and arterial oxygen saturation was assessed utilizing MouseOx Plus 015001 (STARR, USA). Animals were euthanized on 6 h, 1-day, 3-days, 5-days, and 7-days post-instillation for sera and tissues processing. Bronchoalveolar lavage fluid (BALF) was obtained on 3-days post-instillation by cannulating the trachea and lavaging the lungs with 1 mL of cold PBS. For immunological analysis, mice were euthanized on 14-days post-instillation of 2 × 10^^6^ PFU of PA-S, and sera, inguinal lymph nodes, and spleens were collected for further study. In one experiment, dexamethasone was intraperitoneally administered to mice after 30 min of instillation of 2 × 10^^6^ PFU of PA-S. Weight was monitored daily, and mice were euthanized on 3-days post-instillation for lung processing.

To establish live SARS-CoV-2-infected *hACE2* mouse model, the *hACE2* mice were intratracheally instilled with 4 × 10^^5^ PFU of live SARS-CoV-2. The vehicle control received an equal volume of PBS. Weight was monitored daily. Animals were euthanized on 5-days post-instillation for tissues processing.

### Total protein and LDH measurement in BALF

Total protein levels were detected using a BCA method. LDH levels were performed using a CytoTox 96 non-radioactive cytotoxicity assay kit (Promega, USA) following the manufacturer’s instructions. All procedures were carried out in the dark.

### Evans blue dye extravasation

To assess the pulmonary permeability, mice were intravenously injected with 100 µl of 1% Evans Blue in PBS. After 30 min, mice were euthanized and perfused with ice-cold PBS through the right ventricle before lung dissection. Lungs were excised and photographed.

### Cytokine screening and analysis

BALF and sera were collected from mice at indicated points. A customized Luminex Mouse Cytokine 23-plex (IL-1α, IL-1β, IL-2, IL-3, IL-4, IL-5, IL-6, IL-9, IL-10, IL-12p40, IL-12p70, IL-13, IL-17A, Eotaxin, G-CSF, GM-CSF, IFN-γ, MCP-1, MIP-1α, MIP-1β, RANTES, KC and TNF-α, Bio-Rad) was used to screen cytokines in 50 µL of BALF and 1:5 diluted serum samples according to the manufacturer’s instructions.

### Histology and immunofluorescence

Lungs were fixed in 4% paraformaldehyde (PFA) for 2 days at RT, embedded in paraffin, and sectioned at 3 µm. Haematoxylin and eosin (H&E) staining was used to assess pulmonary pathologies. A 5-grade scoring system was adopted to describe the severity of the lung damage from least severe to most severe as described previously.^20^ TUNEL assay was used to assess apoptotic cells in lung tissues via a DeadEndTM Fluorometric TUNEL System (Promega, USA).

Paraffin-embedded sections were incubated with 3% H_2_O_2_ to block endogenous peroxidases and then subjected to an EDTA buffer for antigen retrieval. The sections were then incubated with blocking buffer (5% goat serum). Primary antibodies used for immunohistochemistry or immunofluorescence analysis included rabbit anti-myeloperoxidase (anti-MPO, Abcam, ab9535), rabbit anti-CD4 (Abcam, ab183685), rabbit anti-CD8α (CST, 98941), and rabbit anti CD103 (Abcam, ab224202).

### Cell staining for flow cytometry

Mice were euthanized on 3-days post-instillation of 2 × 10^^6^ PFU of PA-S for lungs and haemocytes processing. Briefly, 100 ul of blood was collected retroorbitally, blended with red blood cell lysis buffer, incubated for 5 min, and then centrifuged at 400 × g for 5 min for haemocytes collection. Lung tissues were dissected from mice, minced on ice into small pieces of less than 1 mm^3^ and then suspended in a 10 mL of digestion buffer consisting of collagenase I (1 mg/mL, Thermofisher, USA), collagenase IV (0.5 mg/mL, Thermofisher) and DNase I (40 U/mL, KeyGen biotech) in DMEM/F12 medium (Gibco, USA). The digestion buffer was incubated with frequent agitation at 37 °C for 50 min, and manual dispersion with 5 mL-pipette-tip every 5 min. Subsequently, an equal volume of DMEM/F12 supplemented with 10% FBS, 1 U/mL streptomycin and 1 U/mL penicillin (Gibco, USA) was added into single-cell suspensions, passed through a 70 µm nylon mesh filter (Corning, USA) and centrifuged at 400 × g for 5 min. All pelleted cells were resuspended into ice-cold PBS supplemented with 0.05% BSA. Throughout the preparation procedure, cells were maintained on ice whenever possible. Besides, mice on 14-days post-instillation of 2 × 10^^6^ PFU of PA-S were also euthanized for inguinal lymph nodes and spleens processing. Cells were collected from inguinal lymph nodes and spleens by grinding and passing throng a 70 µm nylon mesh filter (Corning, USA).

To investigate cell-mediated immune responses, mice were euthanized on 14-days post-instillation of 2 × 10^^6^ PFU of PA-S for isolation of lymphocytes in spleens. Isolated lymphocytes were cultured in RPMI 1640 medium supplemented with 10% heat-inactivated FBS, 100 U/mL penicillin/streptomycin, 1 mM pyruvate (all from Gibco, USA), 50 μM β-mercaptoethanol and 20 U/mL IL-2 (all from Sigma-Aldrich, USA). Simultaneously, RBD, S1, or S protein (5 μg/mL) was added to activate cells (1 × 10^6^ per well) followed by 72 h of incubation at 37 °C. Terminally, the cells were collected for flow cytometric analysis of IL-4 and IFN-γ cytokines levels.

For surface staining, 5 × 10^5^ cells were stained with the indicated antibodies at 4 °C. For intracellular staining, cells were then fixed using the Cytofix solution (BD Biosciences, USA), and incubated with fluorochrome-labeled antibodies specific for the mouse. Antibodies used for flow cytometry analysis included PerCP-Cy5.5 anti-CD45, FITC or Pacific Blue anti-CD11b, APC or AmCyan anti-Ly6C, PE or APC anti-Ly6G, PerCP-Cy5.5 anti-CD3, APC anti-CD4, AmCyan anti-CD8, FITC or PE anti-CD69, FITC anti-CD80, Qdot 655 anti-NK1.1, PE anti-Granzyme B, PE-Cy7 or PE anti-IFN-γ, PE anti-CD44, Qdot 705 anti-CD62L and Pacific Blue anti-IL-4 (all from BD Biosciences or eBiosciences, USA).

### ELISA assay

To investigate the serum antibodies against the S1, mice were euthanized on 14-days post-instillation of 2 × 10^^6^ PFU of PA-S for sera collection. Recombinant RBD, S1, and S were performed to coat flat-bottom 96-well plates (ThermoFisher, USA) at a final concentration of 1 μg/ml in 50 mM carbonate coating buffer (pH 9.6) at 4 °C overnight. Then blocking solution containing 1% BSA in PBST was added for 1 h of incubation at RT. Serially diluted serums were added and incubated at 37 °C for 1 h. Antibodies, including goat anti-mouse IgG and anti-mouse IgM horseradish peroxidase (HRP)-conjugated antibody, were diluted 1: 5,000 in blocking solution and added to wells (100 μl/well) for 1 h of incubation at RT. Development was performed using 3,3′,5,5′-tetramethyl biphenyl diamine (TMB) for 10 min of incubation, followed by the stop of reactions by 50 μl/well of 1.0 M H_2_SO_4_ solution. Absorbance was measured at 450 nm using a microplate reader (Biotek, USA).

### Macrophage and monocyte depletion by clodronate liposome injection

A clodronate liposome administration method was performed to selectively deplete macrophages and monocytes, including pulmonary macrophages.^39^ Clophosome-A was performed following the manufacturer’s instructions. Briefly, 200 μl of clophosome-A or control liposomes were intravenously injected into mice 24 h before the instillation of 2 × 10^^6^ PFU of PA-S and every 2 days thereafter to deplete peripheral or pulmonary interstitial macrophages/monocytes. 50 μl of Clophosome-A or control liposomes was administrated to the lung of mice by pharyngeal aspiration 24 h before the instillation of 2 × 10^^6^ PFU of PA-S to deplete pulmonary resident macrophages, i.e., alveolar macrophages. The clophosome-A method depletes more than 90% of macrophages in the lungs 24 h after injection, an effect that persists for up to 2 days (Supplementary Fig. S6).

### Spike S1vaccine formulation and vaccinations of mice

Spike S1 recombinant protein (S1-WT, aa:16–685) was purchased from Sino Biological with a purity of more than 90%. The recombinant protein vaccine was prepared by mixing S1-WT with AddaS03 adjuvant (AS03, InvivoGen, France) to be an emulsifiable mixture. We used 6–8-week-old male *hACE2* C57BL/6 mice for immunization. The mice received immunization by intramuscular injection of S1-WT vaccine or PBS on days of 0, 14, and 28. Blood samples were collected on day 35 via eye socket vein and centrifuged at 6000 rpm for 10 min for sera collection. The IgG antibody titer against the S1 protein in sera and pseudovirus neutralization activity of the sera were measured (Supplementary Fig. S7). The isolated sera were injected 24 h before the instillation of 2 × 10^^6^ PFU of PA-S in *hACE2* mice for evaluating the block efficiency of sera vaccinated with S1 protein.

### Pseudovirus neutralization assay

The wild type of SARS-CoV-2 luciferase-expressing pseudoviruses was purchased from Genomeditech, China. The pseudovirus neutralization was performed as described previously.^16^ Briefly, luciferase-expressing pseudovirus was pre-incubated with serially diluted immune sera in 96-well plates for 1 h at 37 °C, following adding the mixture to 293 T/ACE2 cells and then incubating for 48 h to express the reporter gene. The efficiency of viral entry was determined with a firefly luciferase assay. In brief, remove the supernatants of infected cells, then add 50 µl of PBS, 50 µl of lysis reagent from a luciferase kit and luciferase substrate (Promega, USA). Relative light units were performed using a multi-mode microplate reader (PerkinElmer, USA).

### Statistical analysis

All data were analyzed using one-way ANOVA or Student’s unpaired *t*-test (GraphPad InStat Software Inc., CA, USA). Results are presented as the means ± SEM. *P* < 0.05 is considered significant (significance is denoted as follows: *ns*, no significance; *P ≤ 0.05; **P ≤ 0.01; ***P ≤ 0.001; ****P ≤ 0.0001).

## Supplementary information

Supplementary information

## Data Availability

All raw data are available from the corresponding author on reasonable request.
