# Supplementary material for: Inactivated SARS-CoV-2 induces acute respiratory distress syndrome in human ACE2-transgenic mice
Source: Signal Transduct Target Ther. 2021 Dec 24;6:439. doi: 10.1038/s41392-021-00851-6 (PMC8705082; doi:10.1038/s41392-021-00851-6)
Supplement: Supplementary file 1 — Supplementary information [file 41392_2021_851_MOESM1_ESM.docx]

Supplementary Materials for

Inactivated SARS-CoV-2 induces acute respiratory distress syndrome in human *ACE2*-transgenic mice

Zhenfei Bi, Weiqi Hong, Haiying Que, Cai He, Wenyan Ren, Jingyun Yang, Tianqi Lu, Li Chen, Shuaiyao Lu, Xiaozhong Peng, Xiawei Wei

Correspondence to: xiaweiwei@scu.edu.cn; pengxiaozhong@pumc.edu.cn; lushuaiyao-km@163.com

**This PDF file includes:**

Figures. S1 to S7

**Figure. S1**


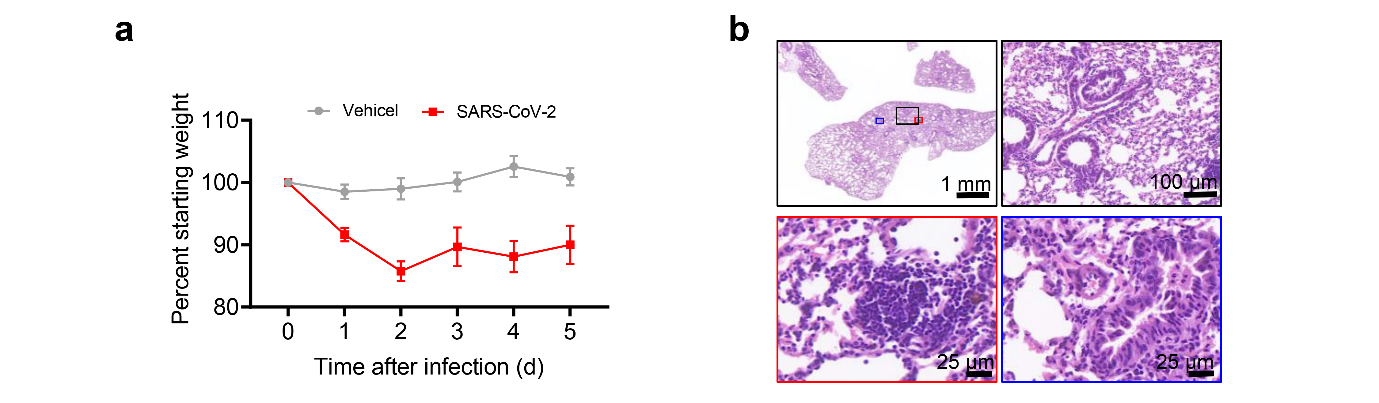


**Live SARS-CoV-2 infection in *hACE2* mice. a**, **b** *hACE2* mice were intratracheally instilled with 4×10^^5^ PFU of SARS-CoV-2. Body-weight changes were monitored (**a**) and pathologies were evaluated by H&E staining (**b**). The magnified images were indicated which area was extracted from the image with low magnification by the individual color border, respectively.

**Figure. S2**


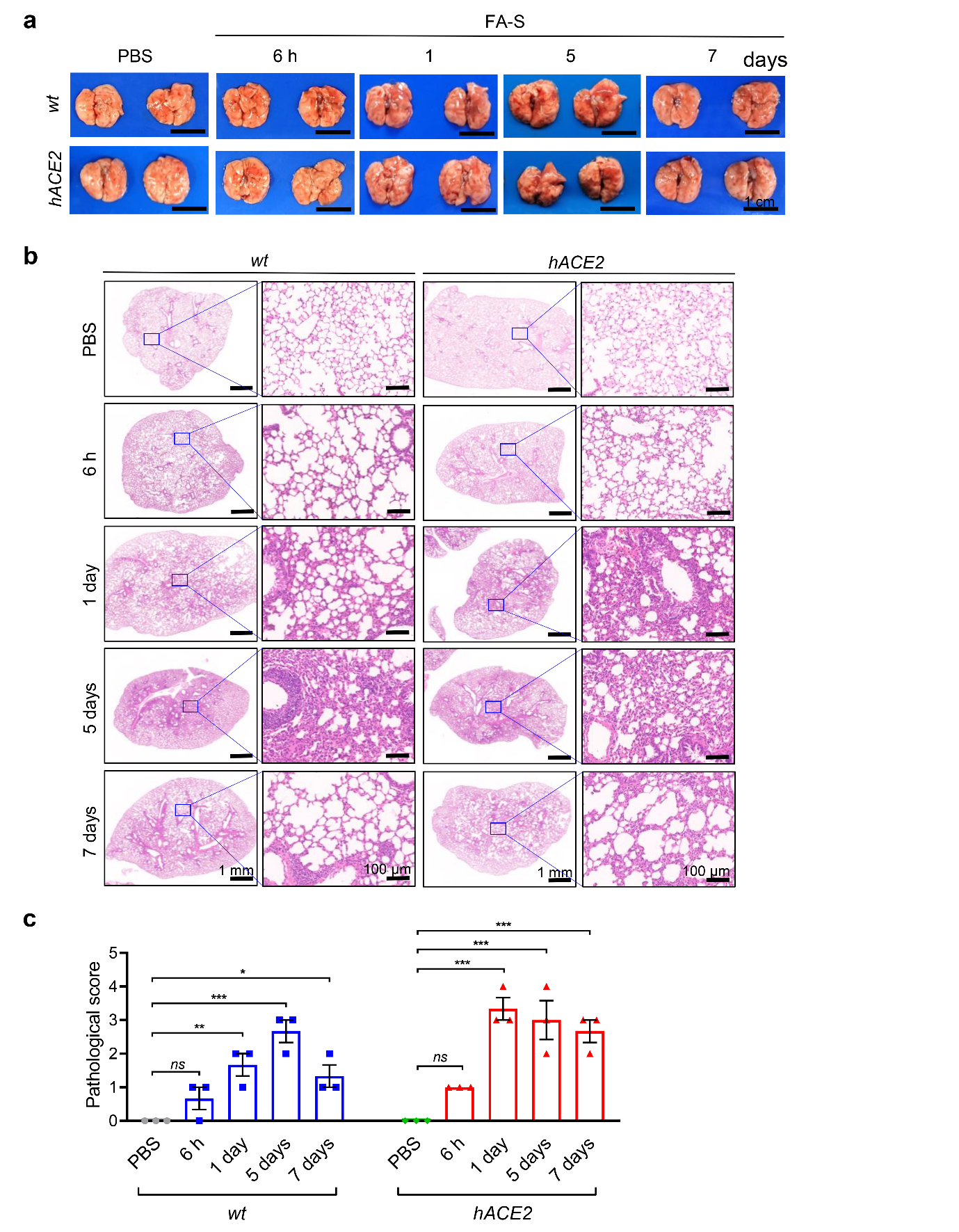


**Pulmonary pathology changes induced by inactivated SARS-CoV-2.** **a-c** Images of lung tissues (**a**) and H&E staining (**b**) and pulmonary pathological scores for mice according to the scoring system (**c**). Data represent the mean ± SEM. Significance is indicated by: *ns*, no significance; **P* ≤ 0.05, ***P* ≤ 0.01, ****P* ≤ 0.001.

**Figure. S3**


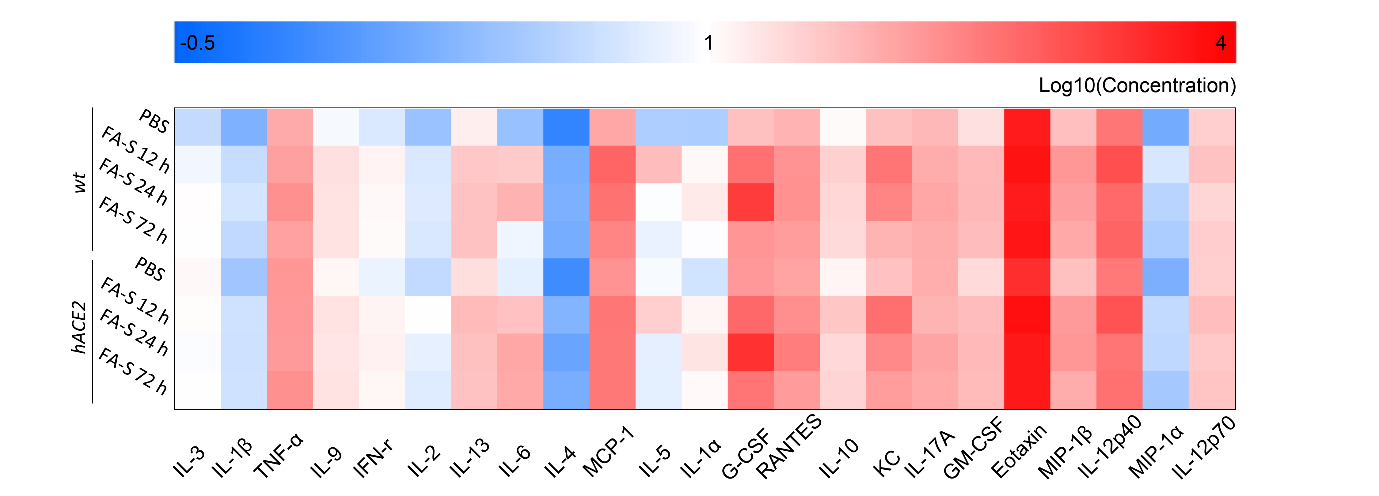


**Screening and analysis of cytokines or chemokines in sera by a customized Luminex Mouse Cytokine 23-plex.** The mice treated with inactivated SARS-CoV-2 showed higher levels of cytokines production on 1 dpi. Results were presented as log10 of the concentration.

**Figure. S4**


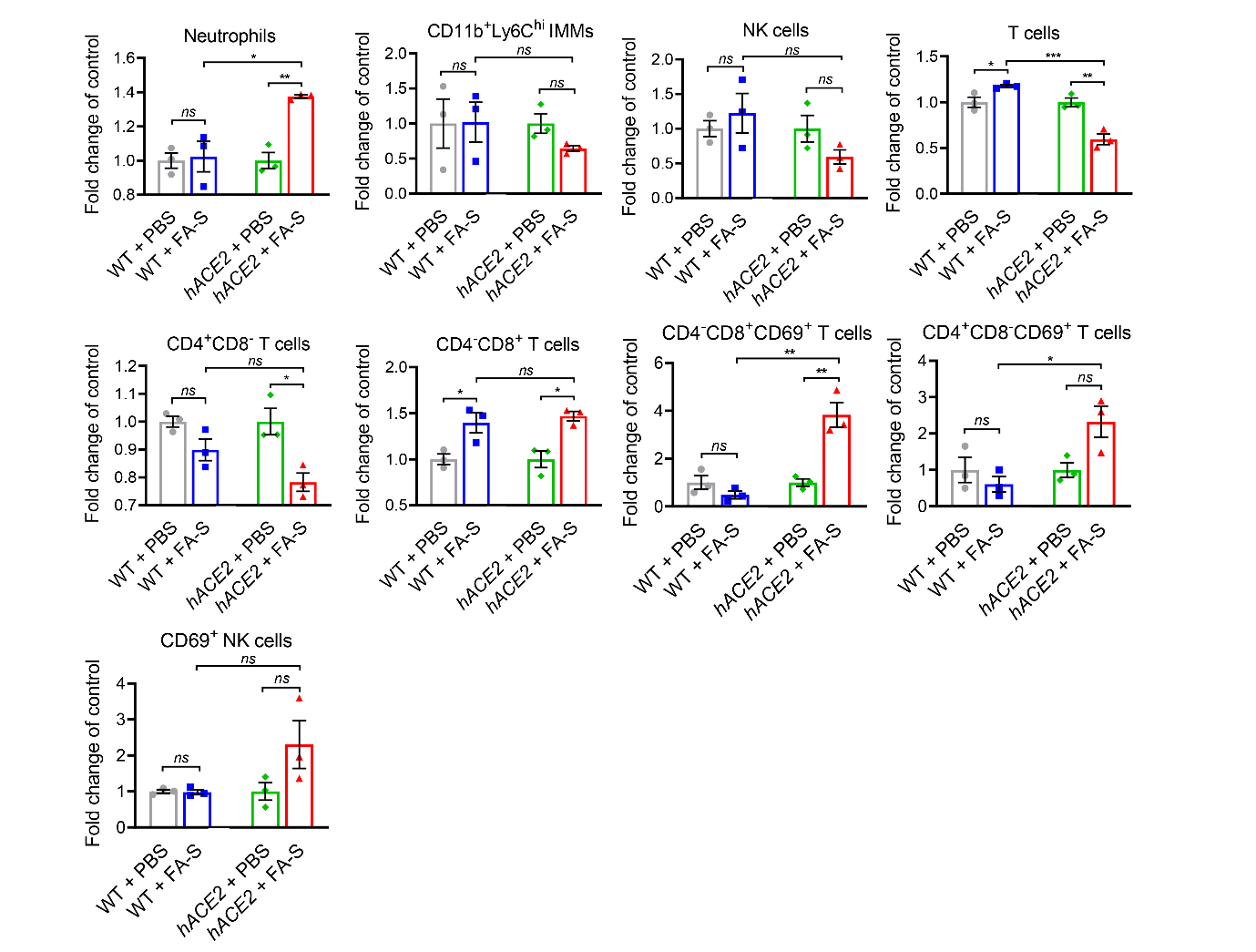


**Flow cytometric analysis of immune response to inactivated SARS-CoV-2 in sera.** Analysis of neutrophils (labelled as Ly6G^+^), inflammatory monocyte-macrophages (IMMs, labelled as CD11b^+^ Ly6C^hi^), NK cells (labelled as NK1.1^+^ CD3^-^), T cells (labelled as CD3^+^ NK1.1^-^) in sera and correlated levels of activation markers (CD69) on 3 dpi. The percentage of CD8-positive T cells were markedly increased in CD3-positive T cells. Lymphopenia was found in *hACE2* mice. Data represent the mean ± SEM. Significance is indicated by: *ns*, no significance; **P* ≤ 0.05, ***P* ≤ 0.01, ****P* ≤ 0.001.

**Figure. S5**


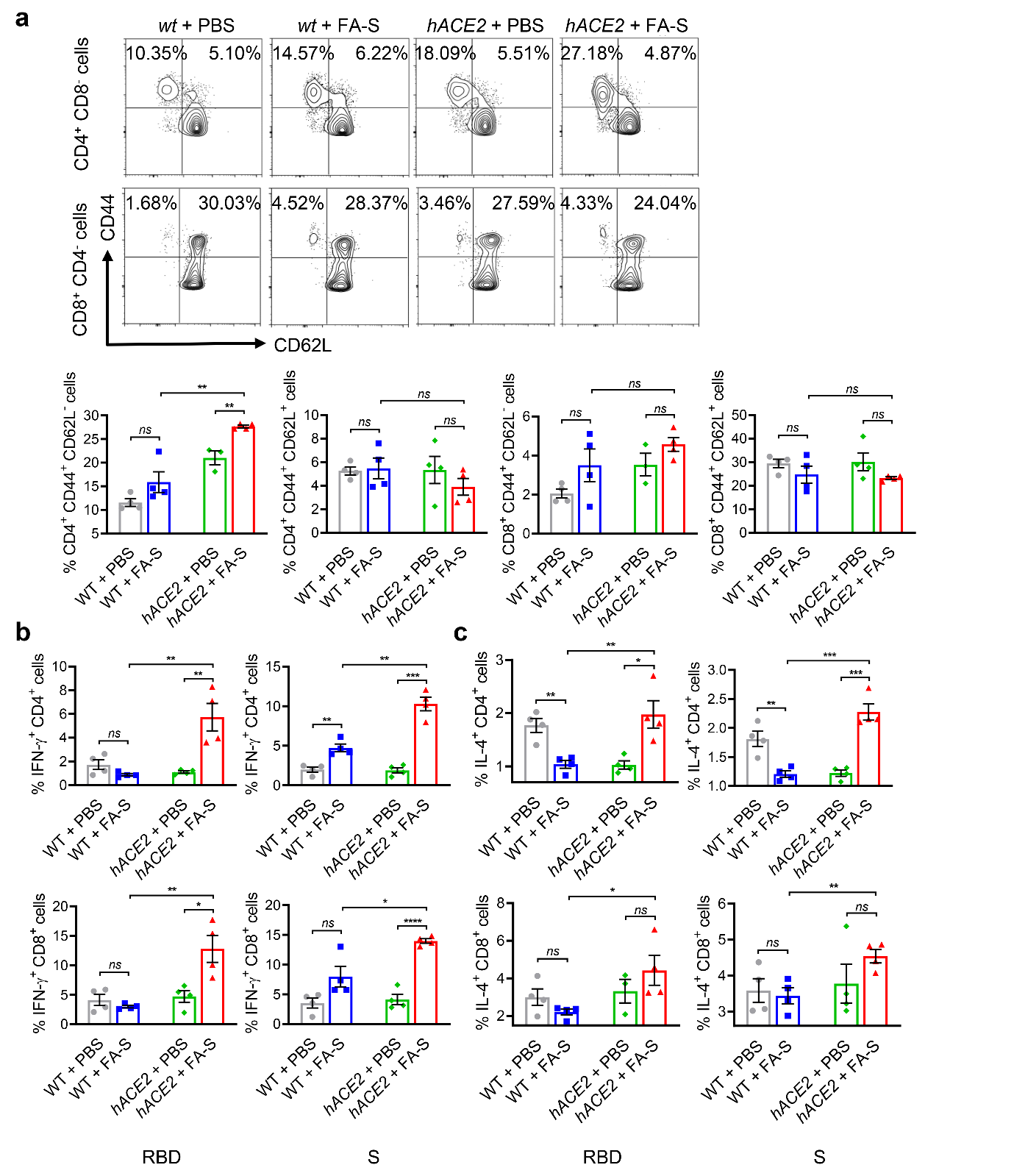


**Analysis of T cell-response from spleens in *hACE2* mice instilled with inactivated SARS-CoV-2.** **a** Flow cytometric analysis of subsets of effector (labeled as CD44^+^ and CD62L^-^) and central (labeled as CD44^+^ and CD62L^+^) memory T cells from spleens on 14 dpi. **b**, **c** Flow cytometric analysis on the secretion of IFN-γ (**b**) and IL-4 (**c**) in T cells from spleens on 14 dpi when stimulated with recombinant RBD or S protein. Data represent the mean ± SEM. Significance is indicated by: *ns*, no significance; **P* ≤ 0.05, ***P* ≤ 0.01, ****P* ≤ 0.001, *****P* ≤ 0.0001.

**Figure. S6**


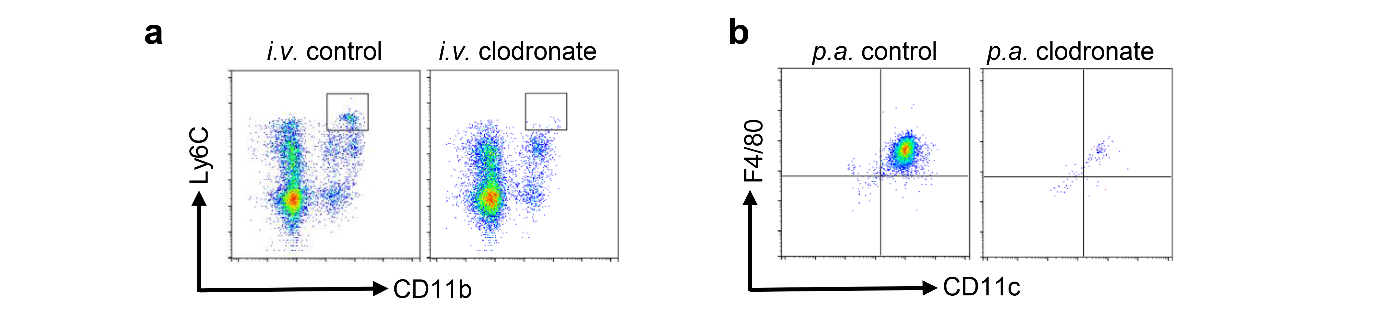


**Flow cytometric analysis of elimination efficiency of clophosome-A. a** Elimination of IMMs by *i.v.* injection of clophosome-A at 24 h. **b** Elimination of pulmonary resident macrophages by *p.a.* administration of clophosome-A at 24 h. *i.v.*, intravenous; *p.a.*, pharyngeal aspiration. Empty liposome was used as a control liposome.

**Figure. S7**


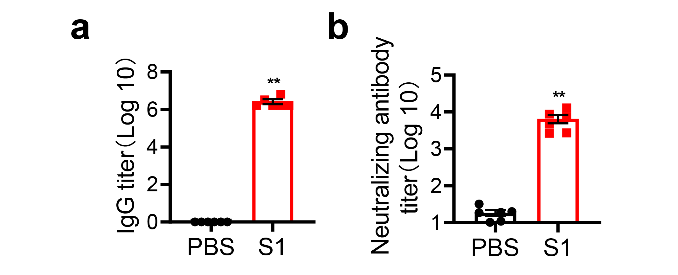


**Identification of antibodies against the subunit 1 of the spike protein and neutralizing antibodies against SARS-CoV-2 pseudovirus in serum from mice immunized with S1 protein. a**, **b** Detection of IgG antibody titer in sera against the S1 protein (**a**) and pseudovirus neutralization activity (**b**) of the sera on 35 days. PBS was used as a control vehicle. Data represent the mean ± SEM. Significance is indicated by ***P* ≤ 0.01.
